# Biallelic Loss‐of‐Function *NDUFA12* Variants Cause a Wide Phenotypic Spectrum from Leigh/Leigh‐Like Syndrome to Isolated Optic Atrophy

**DOI:** 10.1002/mdc3.13398

**Published:** 2022-01-03

**Authors:** Francesca Magrinelli, Elisa Cali, Vinícius Lopes Braga, Uluç Yis, Hoda Tomoum, Hanan Shamseldin, Julian Raiman, Christoph Kernstock, Flávio Moura Rezende Filho, Orlando Graziani Povoas Barsottini, Robert W. Taylor, Elsebet Østergaard, Abdullah Tamim, Karin Schäferhoff, Juliana Maria Ferraz Sallum, Maha S. Zaki, Fernando Kok, Kailash P. Bhatia, Bernd Wissinger, Kate Sergeant, Tobias B. Haack, Rita Horvath, Semra Hiz, Fowzan S. Alkuraya, Henry Houlden, José Luiz Pedroso, Reza Maroofian

**Affiliations:** ^1^ Department of Clinical and Movement Neurosciences, UCL Queen Square Institute of Neurology University College London London United Kingdom; ^2^ Department of Neurosciences, Biomedicine and Movement Sciences University of Verona Verona Italy; ^3^ Department of Neuromuscular Diseases UCL Queen Square Institute of Neurology, University College London London United Kingdom; ^4^ Department of Neurology Universidade Federal de São Paulo São Paulo Brazil; ^5^ Division of Pediatric Neurology, Department of Pediatrics Dokuz Eylül University Faculty of Medicine İzmir Turkey; ^6^ Department of Pediatrics Ain Shams University Cairo Egypt; ^7^ Department of Genetics King Faisal Specialist Hospital and Research Center Riyadh Saudi Arabia; ^8^ Department of Inherited Metabolic Disease Birmingham Children's Hospital Birmingham United Kingdom; ^9^ Center for Ophthalmology Institute for Ophthalmic Research, University of Tübingen Tübingen Germany; ^10^ Wellcome Centre for Mitochondrial Research, Translational and Clinical Research Institute, Faculty of Medical Sciences Newcastle University Newcastle upon Tyne United Kingdom; ^11^ Department of Clinical Genetics Copenhagen University Hospital Rigshospitalet Copenhagen Denmark; ^12^ Department of Clinical Medicine University of Copenhagen Copenhagen Denmark; ^13^ Department of Pediatric Neurology King Faisal Specialist Hospital and Research Center Riyadh Saudi Arabia; ^14^ Institute of Human Genetics and Applied Genomics University of Tübingen Tübingen Germany; ^15^ Department of Ophthalmology Universidade Federal de São Paulo (UNIFESP) São Paulo Brazil; ^16^ Clinical Genetics Department, Human Genetics and Genome Research Institute National Research Centre Cairo Egypt; ^17^ Department of Neurology Universidade de São Paulo (USP) São Paulo Brazil; ^18^ Mendelics Genomic Analysis São Paulo Brazil; ^19^ Oxford Genetics Laboratories Oxford University Hospitals NHS Foundation Trust Oxford United Kingdom; ^20^ Centre for Rare Diseases University of Tübingen Tübingen Germany; ^21^ Department of Clinical Neurosciences University of Cambridge, John Van Geest Cambridge Centre for Brain Repair Cambridge United Kingdom; ^22^ Department of Translational Genomics Center for Genomic Medicine, King Faisal Specialist Hospital and Research Center Riyadh Saudi Arabia; ^23^ Department of Anatomy and Cell Biology College of Medicine, Alfaisal University Riyadh Saudi Arabia

**Keywords:** *NDUFA12*, dystonia, optic atrophy, Leigh syndrome, phenotypic heterogeneity

## Abstract

**Background:**

Biallelic loss‐of‐function *NDUFA12* variants have hitherto been linked to mitochondrial complex I deficiency presenting with heterogeneous clinical and radiological features in nine cases only.

**Objectives:**

To fully characterize, both phenotypically and genotypically, *NDUFA12*‐related mitochondrial disease.

**Methods:**

We collected data from cases identified by screening genetic databases of several laboratories worldwide and systematically reviewed the literature.

**Results:**

Nine unreported *NDUFA12* cases from six pedigrees were identified, with presentation ranging from movement disorder phenotypes (dystonia and/or spasticity) to isolated optic atrophy. MRI showed basal ganglia abnormalities (*n* = 6), optic atrophy (*n* = 2), or was unremarkable (*n* = 1). All carried homozygous truncating *NDUFA12* variants, three of which are novel.

**Conclusions:**

Our case series expands phenotype–genotype correlations in *NDUFA12*‐associated mitochondrial disease, providing evidence of intra‐ and inter‐familial clinical heterogeneity for the same variant. It confirms *NDUFA12* variants should be included in the diagnostic workup of Leigh/Leigh‐like syndromes – particularly with dystonia – as well as isolated optic atrophy.

*NDUFA12* is a nuclear gene encoding the supernumerary subunit A12 of mitochondrial complex I (CI; NADH:ubiquinone oxidoreductase), the foremost multimeric enzyme of the respiratory chain which contributes ~40% of the proton driving force for ATP synthesis.^1^ Subunit A12 is proposed to act in assembling and stabilizing the extramembrane arm of CI.^2^

The first reported case of *NDUFA12*‐related mitochondrial disease was a Pakistani child with Leigh syndrome (LS) carrying a homozygous nonsense variant.^1^ Few *NDUFA12* cases have been described since then, with clinical manifestations ranging from complex neurological syndromes with prominent dystonia/spasticity and MRI evidence of basal ganglia (BG) changes to isolated optic atrophy (OA) in one case.^3^, ^4^

We report nine additional cases from six kindreds (Fig. 1A), all carrying biallelic *NDUFA12* variants, three of which are novel, and review cases previously reported.

**FIG. 1:**
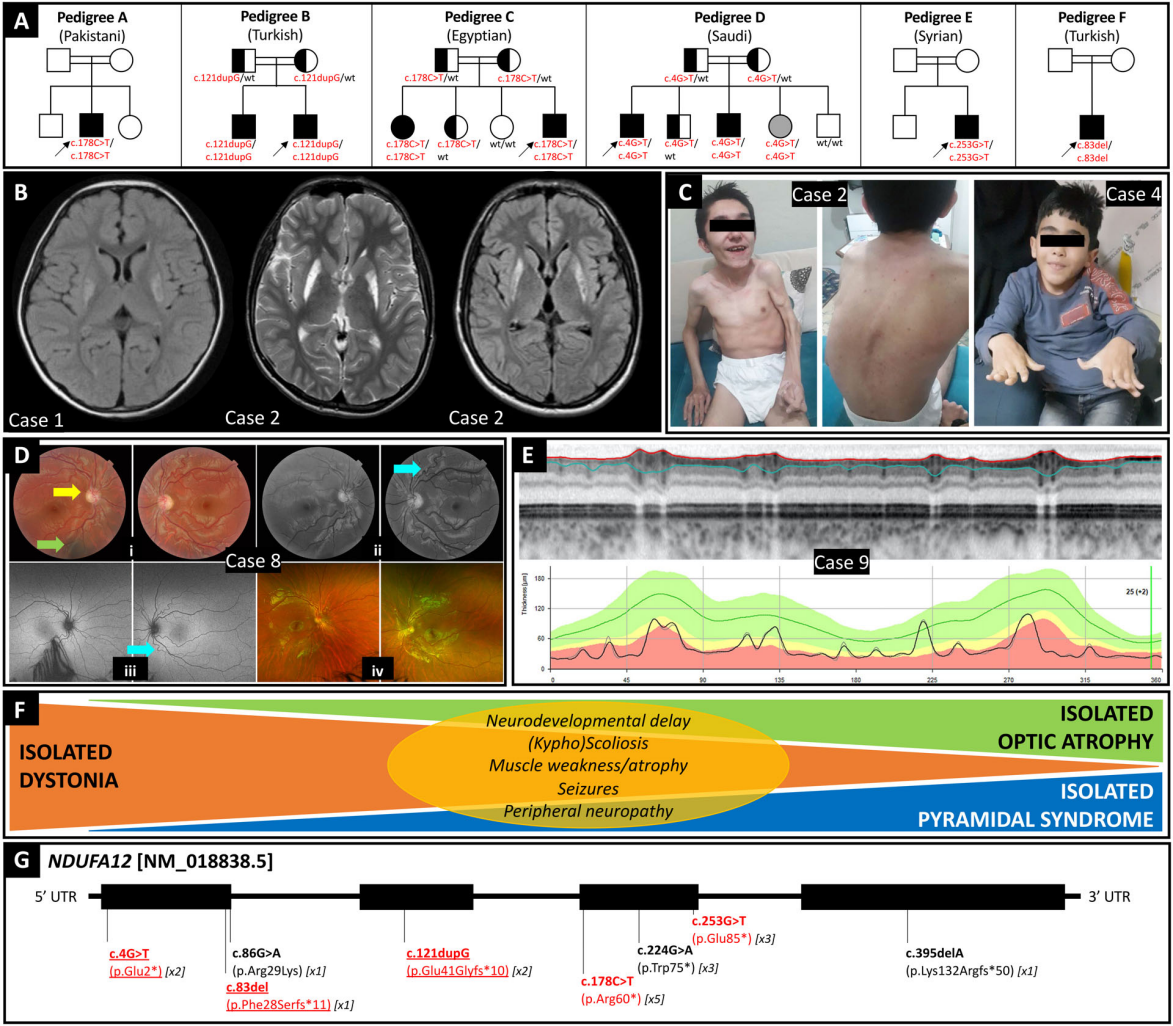
Overview of *NDUFA12*‐associated phenotype–genotype correlations. (A) Family trees of nine new cases herein reported and their ethnicity. Arrows identify probands. Symbols filled in with black and gray indicate homozygotes for the mutant allele who are symptomatic and asymptomatic, respectively. Half‐filled symbols represent asymptomatic heterozygous carriers of the mutant allele; wt = wild type. (B) Brain MRI of Case 1 (left, T2‐FLAIR sequence) showing hyperintense signal of the bilateral lenticular nucleus, and Case 2 (middle, T2 sequence; right, T2‐FLAIR sequence). (C) Video frames of Case 2, highlighting dystonic‐pyramidal features, kyphoscoliosis, and generalized muscle atrophy, and Case 4, showing dystonic involvement of hands and trunk as well as kyphoscoliosis. (D) Case 8: (i) Color fundus oculi showing pale optic discs and decentralized excavation with narrow temporal rim on the left eye (yellow arrow); arterial tortuosity is seen in both eyes and a choroidal nevus can be found on the right eye (green arrow). (ii) Red free photos that highlight the arteriolar tortuosity (light blue arrow). (iii) Panoramic fundus oculi picture depicts no retinal abnormalities. (iv) Normal fundus oculi autofluorescence in both eyes. (E) Case 9: Optical coherence tomography (OCT) showed markedly reduced thickness of the peripapillary nerve fiber layer. (F) Schematic of the wide phenotypic spectrum associated with biallelic loss‐of‐function variants in *NDUFA12*, including dystonia, pyramidal signs, and optic atrophy, either isolated or in different combinations, and additional less prevalent features (yellow oval). (G) Schematic of the *NDUFA12* gene with variants hitherto reported, including those reported in the present case series (highlighted in red; novel variants also underlined). The number of symptomatic subjects carrying the variant reported so far is indicated in squared brackets.

## Methods

To fully characterize the clinical spectrum and course of *NDUFA12*‐related mitochondrial disease, we collected phenotypic and genetic data from cases identified by retrieving databases of several diagnostic and research genetic laboratories worldwide. Variants were prioritized according to the following criteria: (1) variant in *NDUFA12* coding regions or at exon‐intron boundaries; (2) gnomAD v3.1 frequency < 0.001; (3) no phenotype restriction. Among resulting variants, we selected pathogenic and likely pathogenic variants according to the American College of Medical Genetics and Genomics (ACMG) standards and guidelines,^5^ in particular for variants with unequivocal or highly predicted loss‐of‐function effect. A systematic literature review of *NDUFA12* cases previously reported was performed by consulting PubMed® and Google Scholar using “NDUFA12” as search term on 31/05/2021. Predetermined clinical and genetic features were extracted and tabulated. Finally, genes associated with both dystonia and optic neuropathy were identified through Genomics England PanelApp (https://panelapp.genomicsengland.co.uk/) and searched on PubMed® using the gene name as search term on the same date. PubMed® results were filtered for article type “review” and/or “systematic review”. “Mutation” and “patient” were occasionally used as additional search terms to rule out non‐clinical studies. Phenotypes associated with the above‐mentioned genes were outlined to delineate the differential diagnosis of *NDUFA12*‐associated mitochondrial disease.

## Results

### Clinicogenetic Characterization of New Cases

#### Pedigree A

Case 1 This 10‐year‐old Pakistani female, born to healthy consanguineous parents, was delivered at 37 weeks following labor induction due to intrauterine growth restriction (birth weight: 1.9 kg). She had unremarkable developmental milestones. She experienced a febrile seizure at age 4 and developed progressive gait and posture impairment as well as left arm weakness since age 6.5, when brain MRI revealed symmetrical T2‐hyperintensity of the posterior putamen (Fig. 1B), with lesions demonstrating a mixed pattern of chronic gliosis and more acute features of cytotoxic oedema. She experienced an episode of status dystonicus at age 8. One year later, she was admitted to the hospital due to an episode of prolonged lethargy which was ultimately attributed to accidental baclofen overdosing. Paired lactate values in CSF and blood were normal. Repetitive nerve conduction studies (NCS)/electromyography (EMG) were unremarkable. Muscle biopsy revealed non‐specific mild predominance of slow fibers and mild myopathic features, whereas assessment of respiratory chain enzymes (RCE) showed low CI activity (0.034; normal: 0.104–0.268) and normal activity of complexes II, III, and IV. Genetic testing for common mitochondrial and nuclear genes associated with Leigh syndrome came back to be normal. She was started on coenzyme Q10, thiamine, biotin, and anticholinergics and lost to follow‐up between the age of 7 and 10 years. On examination at age 10, she was wheelchair bound with scoliosis and truncal hypotonia, limb flexor spasticity and dystonic posturing of the extremities. Her cognitive functions were unremarkable. Due to recent deterioration of her visual function in the absence of any identifiable traumatic, inflammatory, or infectious trigger, the patient underwent an ophthalmological assessment and was diagnosed with significant visual deficit and severe OA. An extensive next‐generation sequencing panel for nuclear mitochondrial genes revealed a homozygous NM_018838.5(*NDUFA12*):c.178C > T (p.Arg60*) variant.

#### Pedigree B

Case 2 This 21‐year‐old Turkish male, product of a third‐degree consanguineous marriage, developed progressive kyphoscoliosis and gait difficulty with left foot in‐turning since age 7. Dystonia did not respond to levodopa or anticholinergics and became generalized over 2 years. The patient was wheelchair bound at age 11 and experienced intractable focal seizures since age 12. Although there was no history of intellectual disability, he showed cognitive deterioration with disease progression. On examination, he showed generalized dystonia, with dysarthria and feeding difficulty due to oromandibular involvement, kyphoscoliosis, left hand clenching, lower‐limb hyperreflexia, and diffuse muscle atrophy (Fig. 1C; Video 1). There was no visual impairment.

Case 3 Case 2's 25‐year‐old brother had a 15‐year history of tiptoe walking on the left foot, with eversion aggravated by walking (Video 1). Lower‐limb hyperreflexia and kyphoscoliosis were also detected. His cognitive functions were normal, and he graduated from university.

**Video 1:** ***Segment 1*.** Case 2 showed generalized dystonia with severe kyphoscoliosis, left hand clenching, and diffuse muscle atrophy. ***Segment 2*.** Case 2's brother (Case 3) presented with tiptoe walking on the left foot, with eversion aggravated by walking, and severe kyphoscoliosis. ***Segment 3*.** Case 4 showed kyphoscoliosis and acral dystonia with mild dystonic hand tremor. ***Segment 4*.** Case 17 (Table 1) showed tiptoe walking, which worsens with fatigue, particularly on the left.^3^ ***Segment 5***. Case 17's sister (Case 18; Table 1) showed a dystonic‐spastic syndrome, mainly affecting the left hemibody.^3^

Both siblings had no visual impairment nor sensory deficits suggesting peripheral neuropathy. Metabolic workup, including blood lactate and pyruvate, ammonia, urinary organic acids, serum and urine amino acids, and very long chain fatty acids, was unremarkable in both brothers. Their brain MRI showed symmetrical T2‐hyperintensity and atrophy of lentiform nuclei (Fig. 1B), with diminished N‐acetylaspartate peak and normal lactate peak on magnetic resonance spectroscopy (MRS). Whole‐exome sequencing (WES) revealed a homozygous NM_018838.5(*NDUFA12*):c.121dupG (p.Glu41Glyfs*10) variant.

#### Pedigree C

Case 4 This 9‐year‐old Egyptian male, born to first cousins, had normal development until age 2, when he started walking on his tiptoes and falling. Achilles tenotomy surgery provided transient improvement. After age 4, he developed right hand tremor, scoliosis, and progressive stiffness in his lower limbs, with loss of independent walking. He was reported having school problems, but he had never undergone intelligence testing nor shown any deterioration of his cognitive function on follow‐up. On examination, he showed kyphoscoliosis, acral dystonia with dystonic hand tremor, and lower‐limb spasticity (Fig. 1C; Video 1). His visual function was unremarkable. Serum ceruloplasmin, creatine kinase, and amino acids and acylcarnitines were normal. He had increased urine lactate. Brain MRI revealed T2/FLAIR‐hyperintensity and T1‐hypointensity with cystic areas in the BG (Fig. 1B).

Case 5 One of Case 4's elder sisters developed right hand grip weakness since age 16. Her past medical history was otherwise unremarkable, including absence of cognitive and visual issues. Brain MRI showed bilateral T2/FLAIR‐hyperintensity of globi pallidi.

WES identified a homozygous NM_018838.5(*NDUFA12*):c.178C > T (p.Arg60*) variant in both siblings.

#### Pedigree D

Case 6 This 16‐year‐old Saudi male, son of first cousins, experienced motor developmental delay and progressive gait unsteadiness with frequent falls since age 2. He developed fixed flexion of the right hand which progressed to right hemiplegia, and severe visual impairment. He was reported with mild attention deficit disorder, but there was no history of intellectual disability nor cognitive deterioration. Brain MRI detected symmetrical T2‐hyperintensity of the BG, with cystic degeneration on the left. Neurological examination revealed dysarthria and limb spasticity. Ophthalmological assessment revealed OA. Plasma lactate was normal on two occasions, whereas plasma pyruvate was increased (3.07 mg/dl; normal: 0.3–0.7).

Case 7 Case 6's 12‐year‐old brother had a history of severe global neurodevelopmental delay, balance difficulties and falls since age 6. There was no history of intellectual disability nor cognitive impairment. On examination, he was dysarthric and wheelchair bound due to spastic quadriplegia. Brain MRI detected symmetrical T2‐hyperintensity of the BG. Plasma lactate was 4.9 mmol/L (normal: 0.5–1).

WES revealed a homozygous NM_018838.5(*NDUFA12*):c.4G > T (p.Glu2*) variant in both siblings. Segregation analysis revealed that their younger sister, who was asymptomatic and did not present any neurological or ocular manifestations on examination at age 9, was homozygote for the same mutant allele.

#### Pedigree E

Case 8 This 16‐year‐old Syrian male, product of consanguineous parents, had a one‐year history of progressive bilateral visual loss without any identifiable trigger. He had an asymptomatic sibling. On examination, visual acuity (VA) was 20/1600 bilaterally, with normal intraocular pressure and anterior segment US biomicroscopy. Direct and consensual pupillary light reflexes were absent. Fundoscopy disclosed mild optic disc pallor bilaterally, with cup‐to‐disc ratios 0.2 (right eye) and 0.4 (left eye), narrow temporal rim of the left optic disc, and retinal arterial tortuosity bilaterally. Fundus autofluorescence was normal (Fig. 1D). His neurological assessment was otherwise unremarkable, his cognitive functions were normal, and his mood was low. Serum lactate was normal. Serological testing for HIV, syphilis and HTLV was negative. Paraneoplastic antibodies and rheumatological workup including serum anti‐AQP4, anti‐MOG, and anti‐CRMP5 antibodies were unremarkable, as well as serum thiamin, cyanocobalamin, and folate levels. He had normal brain and orbit MRI, and slightly increased CSF protein levels (63.9 mg/dL; normal: <45). WES revealed a homozygous NM_018838.5(*NDUFA12*):c.253G > T (p.Glu85*) variant.

#### Pedigree F

Case 9 This 33‐year‐old Turkish male born to consanguineous parents presented at age 28 with sudden bilateral painless visual loss (VA 20/40), which slowly progressed over the following years. No triggers were identified at symptom onset. There was a history of mild intellectual disability. His VA was 20/200 bilaterally at age 30 and remained relatively stable ever since. Perimetry revealed central scotomas spanning most of the central 30 degrees of the visual field bilaterally, more pronounced in the left eye. Intraocular pressure was normal, and anterior segment US biomicroscopy was unremarkable aside from mild cataract. Pupils were isochoric, with the left one showing relative afferent deficit. Fundoscopy revealed pale optic discs with cup‐to‐disc ratios 0.7 (right eye) and 0.8 (left eye), whereas macula, peripheral retina, and vessels were unremarkable. Bilateral optical coherence tomography detected markedly reduced thickness of the peripapillary retinal nerve fiber layer and microcystic macular edema (left>right; Fig. 1E). Neurological assessment was otherwise unremarkable, with cognitive functions being unchanged since early childhood. Serum lactate levels were not tested. MRI brain orbits detected optic chiasm atrophy. Screening for cardiovascular risk factors, including sleep apnea, was unremarkable. An NGS panel for nuclear and mitochondrial OA‐associated genes detected a heterozygous ENST00000304511.2(*TMEM126A*):c.314G > A (p.Arg105Gln) variant, whose causal relationship was excluded based on high frequency in population databases and in silico prediction tools. Whole‐genome sequencing revealed a homozygous variant NM_018838.5(*NDUFA12*):c.83del (p.Phe28Serfs*11).

### Phenotype–Genotype Characterization of all Reported Cases

Including our series, 18 *NDUFA12* cases (nine males) from 11 pedigrees of Arabian, North African and European ancestry have hitherto been reported (Table 1).^1^, ^3^, ^4^ Perinatal issues were reported in 5/18 (27.8%) patients^3^, ^4^ (one with coexistent mucolipidosis type II).^4^ Neurodevelopmental milestones were delayed in 5/18 (27.8%) cases.^1^, ^3^ Age of symptom onset ranged from birth to 28 years, with acute or subacute gait (10/18 cases, 55.6%) or visual (4/18, 22.2%) impairment being the most frequent presenting features.^1^, ^3^ Main clinical manifestations (Fig. 1F) encompassed dystonia (11/18 cases, 61.1%), pyramidal features (8/18, 44.4%), and visual impairment (7/18, 38.9%), either isolated or combined. (Kypho)Scoliosis (6/18 cases, 33.3%), muscle weakness/atrophy (5/18, 27.8%), sporadic seizures (2/18, 11.1%), and peripheral neuropathy (1/18, 5.6%)^3^ represented minor phenotypic features. Brain MRI was unremarkable in two patients with isolated visual impairment^3^ and revealed BG T2‐hyperintensity, BG atrophy/cystic degeneration, and optic chiasm atrophy in 13/18 (72.2%), 9/18 (50.0%), and 1/18 (5.6%) cases, respectively. Lactate was increased in blood, urine, or CSF in 10/13 (76.9%). NCS documented peripheral neuropathy in one out of two cases where available.^3^ Muscle biopsy, whose details were available in two cases, showed only subtle myopathic features. In five cases, low CI activity was documented on assessment of RCE.^1^ Parental consanguinity was reported in 17/18 (94.4%) cases, with 11/18 (61.1%) having at least one affected sibling. Eight homozygous *NDUFA12* pathogenic variants have been hitherto reported, including three novel variants described here (p.Glu2*, p.Phe28Serfs*11, p.Glu41Glyfs*10). These are scattered throughout *NDUFA12* exons, all but one being truncating variants (4 nonsense, 3 frameshift; Fig. 1G). Intriguingly, the asymptomatic sibling of two affected individuals (Pedigree D) carried the mutant allele in the homozygous state, thus suggesting either incomplete penetrance or further intra‐familial variability with respect to age at symptom onset.^6^

**TABLE 1:** Demographic, phenotypic, and genetic features of nine new and nine previously reported *NDUFA12* cases

Pedigree	Case	Sex/AE (y)	Ethnicity	Consanguinity	FH	Perinatal history/NDM	AO (y)	Symptom(s) at onset	Clinical manifestations			Brain MRI	Findings from other investigations	NDUFA12 variants (NM_018838.5)	Ref
									Neurological	Ophthalmological	Other				
**Cases first reported in this study**															
A	1	F/10	Pakistani	Yes	No	Premature induced birth due to intrauterine growth restriction. Normal NDM.	6.5	Gait abnormality and left arm weakness	Trunk hypotonia; dystonic posturing; episode of status dystonicus.	Visual impairment	Febrile convulsion; scoliosis	T2 hyperintensities of the posterior putamen bilaterally (chronic gliotic scarring and acute cytotoxic oedema).	Optic atrophy. Normal paired plasma and CSF lactate. NCS/EMG: no evidence of peripheral neuropathy. MB: mild predominance of slow fibers. RCE: low complex I, normal complexes II‐III‐IV.	Hom c.178C > T (p.Arg60^§§^)	‐
B^§^	2	M/21	Turkish	Yes	Yes	Normal birth. Normal NDM.	7	Gait disturbance with unilateral intoeing	Generalized dystonia, including oromandibular and left foot dystonia which worsens with exercise; focal seizures (since age 12); cognitive decline.	None	Kyphoscoliosis	Atrophy and T2 hyperintensity of lentiform nucleus	Plasma lactate and pyruvate, ammonia, urinary organic acids, serum and urine amino acids, VLCFA: normal. MRS: neuronal loss in lentiform nucleus.	Hom c.121dupG (p.Glu41Glyfs^§§^10)	‐
B^§^	3	M/25	Turkish	Yes	Yes	Normal birth. Normal NDM.	10	Gait disturbance with unilateral intoeing	Left foot dystonia, which worsens with exercise; hyperreflexia lower limbs.	None	Kyphoscoliosis	Atrophy and T2 hyperintensity of lentiform nucleus bilaterally	Plasma lactate and pyruvate, ammonia, urinary organic acids, serum and urine amino acids, VLCFA: normal. MRS: neuronal loss in lentiform nucleus.	Hom c.121dupG (p.Glu41Glyfs^§§^10)	‐
C^§^	4	M/9	Egyptian	Yes	Yes	Normal birth. Motor developmental delay	2	Unilateral tip‐toe walking, recurrent falls	Right hand tremor; generalized Spasticity lower limbs Muscle weakness	None	Kyphoscoliosis	T2/FLAIR hyperintensity and T1 hypointensity of BG, with some cystic areas	Normal amino acid/acylcarnitine. Increased urine lactate. CK and ceruloplasmin: normal.	Hom c.178C > T (p.Arg60^§§^)	‐
C	5	F/20	Egyptian	Yes	Yes	Normal birth. Normal NDM.	16	Clumsiness and tremor of the right hand	Muscle weakness right hand	None	‐	T2 hyperintensity of GP	‐	Hom c.178C > T (p.Arg60^§§^)	‐
D	6	M/16	Saudi	Yes	Yes	Global delay of NDM	2	Gait unsteadiness and falls Spastic‐dystonic tetraparesis	Right hand dystonia	Visual impairment	‐	Bilateral symmetrical T2 hyperintensity of BG along with left BG encephalomalacia	Optic nerve atrophy. Normal plasma lactate. Increased plasma pyruvate.	Hom c.4G > T (p.Glu2^§§^)	‐
D	7	M/12	Saudi	Yes	Yes	Global delay of NDM	6	Gait unsteadiness and falls	Spastic quadriplegia	None	Recurrent UTI; mild attention deficit disorder.	Bilateral symmetrical T2 hyperintensity of BG	Increased plasma lactate.	Hom c.4G > T (p.Glu2^§§^)	‐
E	8	M/16	Syrian	Yes	No	Normal birth. Normal NDM.	15	Subacute visual loss	Absent direct and consensual pupillary light reflexes	Visual impairment	Depression	Normal	V.A. 20/1600 bilaterally. Optic atrophy. Normal plasma lactate. Serology for HIV, syphilis, HTLV: negative. Autoimmune screening: negative. CSF: mildly elevated proteins (63.9 mg/dL; r.i. <45 mg/dl).	Hom c.253G > T (p.Glu85^§§^)	‐
F	9	M/33	Turkish	Yes	No	Normal birth. Normal NDM.	28	Acute visual loss	None	Visual impairment	‐	Optic chiasm atrophy	V.A. 20/200 (age 30). Optic atrophy. OCT: markedly thickness of peripapillary nerve fiber layer. NGS gene panel for optic atrophy: negative.	Hom c.83del (p.Phe28Serfr^§§^11)	‐
**Cases previously reported**															
G^§§^	10	F/21	Pakistani	Yes	No	Normal birth. Delayed early motor NDM.	2	Regression of motor abilities	Generalized dystonia with dystonic spasms Severe muscle atrophy Slight intellectual disability Dysphagia (PEG) Wheelchair bound	Visual impairment (detected on follow‐up at age 18)	Scoliosis (surgery at age 18) Growth retardation Hypertrichosis Strabismus	T2 hyperintensity of GP	MB: type 1 fiber atrophy and fiber type disproportion. RCE: low complex I, normal complexes II‐III‐IV. Brain MRS: elevated lactate in the whole cerebrum. V.A. 20/25 (right eye), 20/32 (left eye). Fundoscopy: pale optic discs. Plasma lactate: 4.9 mmol/l (r.i. <2.1). CSF lactate: 2.4 mmol/l (r.i. 1.1–1.8). Pale optic discs.	Hom c.178C > T (p.Arg60^§^)	1
H^§§§^	11	F/Birth	Mennonite	No	No	Symmetric intrauterine growth restriction^§§§^	Birth	Facial dysmorphisms, short limbs, persistent thrombocytopenia, direct hyperbilirubinemia, and poor feeding^§§§^	–	‐	Facial dysmorphisms (large ears with increased folding anteriorly, long philtrum, small mouth with prominent alveolar ridge, epicanthal folds), short upper and lower extremities with bowing of the tibia and fibula bilaterally, supinated ankles, and mild generalized hypertonia. Flexion wrist contractures, clenched fists. Thumbs were held between the second and third fingers, bilaterally.^§§§^		Elevated ALP; low serum vitamin D 25‐OH; direct hyperbilirubinemia. US abdomen: normal. Thrombocytopenia. Congenital infection screen: negative. US head: normal. Echocardiogram: patent ductus arteriosus and patent foramen ovale. Chest X‐ray: hypoinflated lungs, dysplastic bones throughout the thorax and visualized upper extremities. Skeletal survey: marked osteopenia, foreshortened long bones with thickened diaphyses, irregular “raggedy” metaphyses, and no wormian bones.^§§§^	Hom c.178C > T (p.Arg60^§^)	4
I	12	M/9	Italian	Yes	No	Birth: poor sucking, hypotonia. Mild global delay of NDM.	4	Sudden‐onset convergent strabismus	Strabismus, nystagmus, minimal ptosis in the left eye	None	None	Age 5: T2 hyperintensity of the brainstem (red nuclei and tegmental tract) Follow‐up MRIs: normal.	Brain MRS: normal. VEP: increased latency in both eyes. ERG: normal. BAEPs: normal. ECG/Ecocardiography: normal. Plasma lactate: 2.85 mmol/l (r.i. 0.5–2.2) on one occasion only. RCE (muscle homogenate): low complex I, normal complexes II‐III‐IV.	Hom c.86G > A (p.Arg29Lys)	3
L	13	F/15	Italian	Yes	No	Birth: respiratory distress. Normal NDM.	6	Dystonia right arm	Generalized dystonia Spastic‐dystonic gait evolving to spastic‐dystonic tetraparesis Oromandibular dystonia	None	Scoliosis	Typical Leigh syndrome pattern. Follow‐up MRI: lesions in the BG (putamen), partial agenesis of septum pellucidum, and mild enhancement of left optic nerve after gadolinium	Brain MRS: normal. VEP: abnormal in amplitude in both eyes. Cardiological evaluation: normal. Plasma lactate: 3.62 mmol/l (r.i. 0.5–2.2). Urine lactate: >400 mmol/l creatinine (r.i. <200). Plasma amino acids: increased alanine (852 mmol/l; r.i. 150–400). RCE (muscle homogenate): low complex I, normal complexes II‐III‐IV.	Hom c.395delA (p.Lys132Argfs^§^50)	3
M	14	F/13	Moroccan	Yes	Yes	Birth: respiratory distress. Normal NDM.	4	Sudden‐onset nystagmus, right hemiparesis	Generalized dystonia Trunk hypotonia Extrapyramidal syndrome Peripheral neuropathy	None	None	T2 hyperintensity of lentiform nucleus and brainstem	Brain MRS: lactate peak. Plasma lactate: 2.4 mmol/l (r.i. 0.5–1.95). CSF lactate: 2.8 mmol/l (r.i. 1–1.9). NCS: peripheral neuropathy. RCE (muscle homogenate): low complex I, normal complexes II‐III‐IV.	Hom c.224G > A (p.Trp75^§^)	3
M	15	F/9	Moroccan	Yes	Yes	Normal NDM.	9	Sudden‐onset visual impairment	Extrapyramidal syndrome	Visual impairment	None	T2 hyperintensity of lentiform nucleus	Hyperlactatorachia (lactate 2.8 mmol/L; r.i. 1–1.90, with normal plasma lactate	Hom c.224G > A (p.Trp75^§^)	3
M	16	F/7	Moroccan	Yes	Yes	Normal NDM.	7	Visual impairment	None	Visual impairment	None	Normal	Optic atrophy. Hyperlactatorachia (2.4 mmol/L; r.i. 1–1.90)	Hom c.224G > A (p.Trp75Ter)	3
N^§^	17	M/11	Syrian	Yes	Yes	Normal NDM.	5	Limb dystonia	Limb dystonia	None	Episodes of vomiting	T2/T2‐FLAIR hyperintensity of lentiform nucleus and red nucleus	FO, VEP, ERG, BAEPs: normal. Serum lactate: 2.6 mmol/L (r.i. 0.5–2.20) Brain MRS: normal.	Hom c.253G > T (p.Glu85^§^)	3
N^§^	18	F/4	Syrian	Yes	Yes	Normal birth. Normal NDM.	3.5	Limb dystonia	Limb and oromandibular dystonia. Rigidity. Tip‐toe walking.	None	None	N.A.	Serum lactate: 2.2 mmol/L (r.i. 0.5–2.20). Acylcarnitine profile and urinary organic acids: normal.	Hom c.253G > T (p.Glu85^§^)	3

Abbreviations: AE, age at last evaluation; ALP, alkaline phosphatase; AO, age of onset; BG, basal ganglia; CSF, cerebrospinal fluid; F, female; FH, family history; FO, fundus oculi; GP, globus pallidus; Hom, homozygote; M, male; MB, muscle biopsy; MRI, magnetic resonance imaging; MRS, magnetic resonance spectroscopy; N.A., not available; NDM, neurodevelopmental milestones; OCT, optical coherence tomography; PEG, percutaneous gastrostomy; Ref, reference; r.i., reference interval; UTI, urinary tract infections; V.A., visual acuity; VLCFA, very long chain fatty acids; Y, years.

§ See also Video 1.

^§§^ Original case reported by Ostergaard et al.^1^ with additional information from an 11‐year follow‐up herein presented.

^§§§^ Co‐occurrence of biallelic variants in *NDUFA12* and biallelic variants in *GNPTAB* (mucolipidosis II alpha/beta).^4^

## Discussion

CI deficiency is the commonest biochemical defect in children with mitochondrial diseases, including LS/Leigh‐like syndrome (LLS).^7^, ^8^, ^9^ Diagnosis of LS requires progressive neurological deterioration with clinical evidence of BG and/or brainstem dysfunction, developmental delay, and elevated serum or CSF lactate, along with neuroradiological or neuropathological evidence of BG and/or brainstem lesions.^8^ When these stringent criteria are not fulfilled (e.g., atypical neuroimaging, normal lactate levels), a diagnosis of LLS can be considered.^8^ Six cases in our series fulfilled the diagnostic criteria for LS/LLS, thus providing further evidence that biallelic loss‐of‐function *NDUFA12* variants are a rare cause of these phenotypes.^1^, ^3^

Dystonia, either isolated or combined with pyramidal features, emerges as the most common motor feature in *NDUFA12*‐related LS/LLS. As dystonia was always associated with MRI evidence of BG damage in our series and previous cases, a secondary etiology (structural damage caused by mitochondrial dysfunction) most likely explains its occurrence in *NDUFA12*‐related LS/LLS.^10^ By contrast, we confirmed that biallelic *NDUFA12* variants are associated with isolated OA, even in the absence of MRI findings, as previously reported in one case.^3^ In the original case reported by Ostergaa*rd et al*.^1^ and Case 1, visual impairment occurred some years after disease onset, thus suggesting visual function should be assessed on follow‐up of patients harboring biallelic *NDUFA12* variants with pure motor presentations. Overall, this adds to the observation that the best‐established nuclear genes linked to OA (Table S1) are involved in mitochondrial pathways, as are pathogenic variants in mitochondrial DNA accounting for Leber hereditary optic neuropathy.^11^ An overview of genes associated with both dystonia and OA is provided in Table S1. Unlike several of these genes, which are recognized with both dominant and recessive inheritance, *NDUFA12* is more likely associated with recessive inheritance only since the observed/expected ratio for both missense and loss‐of‐function variants is close to 1 according to gnomAD. Finally, we highlight that increased lactate levels were detected in only two out eight cases of our new cohort when this testing was performed, whereas plasma pyruvate level was increased in one case with normal plasma lactate. This proportion is lower than in previously reported cases, which is in keeping with a higher prevalence of LLS phenotype in our series.^8^

Our series expands the age of onset of *NDUFA12*‐related mitochondrial disease to as late as 28 years. Furthermore, it demonstrates significant intra‐familial variability (e.g., Cases 2, 3, 4, 5, 6‐7) and occurrence of the same *NDUFA12* variant in patients/kindreds with different phenotypes (inter‐familial heterogeneity, e.g., p.Glu85* associated with OA in Case 8 and LS in Pedigree N). This suggests that yet undetermined genetic, epigenetic, and environmental factors modulate the variable expression of mutant *NDUFA12* alleles at the phenotypic level.^6^

In conclusion, our case series expands the phenotype–genotype spectrum of *NDUFA12*‐associated mitochondrial disease and provides evidence of inter‐ and intra‐familial clinical heterogeneity associated with the same variant. It supports the inclusion of *NDUFA12* variants in the diagnostic workup of not only LS/LLS, particularly when dystonia is the prominent motor manifestation, but also isolated OA.

## Author Roles

(1) Research project: A. Conception, B. Organization, C. Execution; (2) Statistical Analysis: A. Design, B. Execution, C. Review and Critique; (3) Manuscript: A. Writing of the first draft, B. Review and Critique.

FM: 1A, 1B, 1C, 2B, 3A;

EC: 1B, 1C, 3A;

VLB: 1C, 3B;

UY: 1C, 3B;

HT: 1C, 3B;

HS: 1C, 3B;

JR: 1C, 3B;

CK: 1C, 3B;

FMRF: 1C, 3B;

OGPB: 1C, 3B;

RWT: 1C, 3B;

EO: 1C, 3B;

AT: 1C, 3B;

KS: 1C, 3B;

JMFS: 1C, 3B;

MSZ: 1C, 3B;

FK: 1C, 3B;

KPB: 1C, 3B;

BW: 1C, 3B;

KS: 1C, 3B;

TH: 1C, 3B;

RH: 1C, 3B;

SH: 1C, 3B;

FSA: 1C, 3B;

HH: 1C, 3B;

JLP: 1C, 3B;

RM: 1A, 1B, 1C, 3B.

## Disclosures

### Ethical Compliance Statement

We confirm that we have read the Journal's position on issues involved in ethical publication and affirm that this work is consistent with those guidelines. The authors confirm that the approval of an institutional review board was not required for this work. We confirm that we have obtained the patient consent for genetic testing on a research basis as well as for video acquisition and publication.

### Funding Sources and Conflicts of Interest

Biological samples from pedigree C were collected as part of the SYNaPS Study Group collaboration funded by The Wellcome Trust and strategic award (Synaptopathies) funding (WT093205 MA and WT104033AIA) and research was conducted as part of the Queen Square Genomics group at University College London, supported by the National Institute for Health Research University College London Hospitals Biomedical Research Centre.

Biological samples from pedigree D were collected as part of the project RAC# 2121053.

Francesca Magrinelli is supported by the Edmond J. Safra Foundation and by the research grant “Fondo Gianesini” in collaboration with UniCredit Foundation and University of Verona, Italy.

Robert W. Taylor is supported by the Wellcome Centre for Mitochondrial Research (203,105/Z/16/Z), the MRC International Centre for Genomic Medicine in Neuromuscular Disease (MR/S005021/1), Mitochondrial Disease Patient Cohort (UK) (G0800674), the UK NIHR Biomedical Research Centre for Aging and Age‐related disease award to the Newcastle upon Tyne Foundation Hospitals NHS Trust, The Lily Foundation, the Pathology Society and the UK NHS Highly Specialized Service for Rare Mitochondrial Disorders of Adults and Children.

Kailash P. Bhatia has received grant support from Wellcome/MRC, NIHR, Parkinson's UK and EU Horizon 2020.

Tobias B. Haack was supported by the Deutsche Forschungsgemeinschaft (DFG, German Research Foundation)—418081722, 433158657.

Kate Sargeant thanks the UK NHS Specialist Commissioners, which funds the “Rare Mitochondrial Disease Service for Adults and Children” in Oxford for their support. The views expressed are those of the author(s) and not necessarily those of the NHS or the UK Department of Health and Social Care.

Rita Horvath is a Wellcome Trust Investigator (109,915/Z/15/Z), who receives support from the Medical Research Council (UK) (MR/N025431/1 and MR/V009346/1), the European Research Council (309548), the Newton Fund (UK/Turkey, MR/N027302/1), the Addenbrookes Charitable Trust (G100142), the Evelyn Trust, the Stoneygate Trust, the Lily Foundation and an MRC strategic award to establish an International Centre for Genomic Medicine in Neuromuscular Diseases (ICGNMD) MR/S005021/1. This research was supported by the NIHR Cambridge Biomedical Research Centre (BRC‐1215‐20,014). The views expressed are those of the authors and not necessarily those of the NIHR or the Department of Health and Social Care.

Henry Houlden is funded by The MRC (MR/S01165X/1, MR/S005021/1, G0601943), The National Institute for Health Research University College London Hospitals Biomedical Research Centre, Rosetree Trust, Ataxia UK, MSA Trust, Brain Research UK, Sparks GOSH Charity, Muscular Dystrophy UK (MDUK), Muscular Dystrophy Association (MDA USA).

The authors declare that there are no conflicts of interest relevant to this work.

### Financial Disclosures for the Previous 12 Months

Kailash P. Bhatia receives royalties from publication of the Oxford Specialist Handbook Parkinson's Disease and Other Movement Disorders (Oxford University Press, 2008), of Marsden's Book of Movement Disorders (Oxford University Press, 2012), and of Case Studies in Movement Disorders – Common and uncommon presentations (Cambridge University Press, 2017). He has received honoraria/personal compensation for participating as consultant/scientific board member from Ipsen, Allergan, Merz and honoraria for speaking at meetings and from Allergan, Ipsen, Merz, Sun Pharma, Teva, UCB Pharmaceuticals and from the American Academy of Neurology and the International Parkinson's Disease and Movement Disorders Society.

## Supporting information


**Table S1** Overview of disease genes associated with dystonia and optic atrophy which are of interest in the differential diagnosis of *NDUFA12*‐associated mitochondrial disease
