# Supplementary material for: Biallelic Loss‐of‐Function NDUFA12 Variants Cause a Wide Phenotypic Spectrum from Leigh/Leigh‐Like Syndrome to Isolated Optic Atrophy
Source: Mov Disord Clin Pract. 2022 Jan 3;9(2):218–28. doi: 10.1002/mdc3.13398 (PMC8810437; doi:10.1002/mdc3.13398)
Supplement: Supplementary file 1 — Table S1 Overview of disease genes associated with dystonia and optic atrophy which are of interest in the differential diagnosis of NDUFA12‐associated mitochondrial disease [file MDC3-9-218-s001.docx]

**Supplementary Table 1. Overview of disease genes associated with dystonia and optic atrophy which are of interest in the differential diagnosis of NDUFA12-associated mitochondrial disease**

| **Gene** | **MOI** | **Dystonia** | **Movement disorder** | | **Pyramidal signs** | | **Ataxia** | **Muscle/ nerve involvement** | **Seizures** | | **Cognitive impairment/delay** | **Type of ocular involvement** | **Magnetic Resonance Imaging** | **Hearing loss** | **Other findings** |
| --- | --- | --- | --- | --- | --- | --- | --- | --- | --- | --- | --- | --- | --- | --- | --- |
| ***NDUFA12*** | AR | Focal or generalized dystonia. | In some cases, extrapyramidal syndrome | | Spasticity | | No | Muscle atrophy, uncommon | Rare | | Variably present | Common optic atrophy | Hyperintensities | No | Scoliosis, facial dysmorphisms |
|  | | | | | | | | | | | | | | | |
| **Disorders in which dystonia and optic atrophy are prominent manifestations** | | | | | | | | | | | | | | | |
| ***C19orf12****^1^* | AD/AR | Generalized or oromandibular dystonia. | Mild parkinsonism, variable | | Spasticity | | Variably present | Distal muscle weakness, motor axonal neuropathy | No | | Variably present | Bilateral optic atrophy | Iron accumulation, cerebellar atrophy | No |  |
| ***MECR^2^*** | AR | Yes, main phenotype, childhood onset. | Chorea variably present | | Spasticity | | Yes, variable, dysarthria. | One with neck muscle weakness | No | | Rare | Bilateral optic atrophy | Bilateral hyperintense T_2_-weighted signal in one or more structures of the basal ganglia |  | Dysphagia |
| ***TIMM8A^3^*** | XLR | Main phenotype, progressive, adult-onset | Parkinsonism | | Spasticity, variably present | | Variably present | Variable Peripheral neuropathy | No | | Variably present | Either optic atrophy or cortical visual impairment | Symmetrical caudate head atrophy | Yes |  |
| ***NDUFS1****^4^* | AR | Frequent | Common hyperkinetic movements | |  | |  | Uncommon | Variably present | | Frequent | Frequent optic atrophy | White matter changes, basal ganglia involvement |  | Feeding difficulties |
| ***SDHA****^5^* | AR/AD | Frequently associated with biallelic mutations | Myoclonus | | Spasticity | | Cerebellar gait ataxia | Muscle weakness, sensory neuropathy | Yes | | Yes | Optic atrophy (both AR and AD) | Cerebellar atrophy, Leigh-like pattern |  | Cardiomyopathy |
|  | | | | | | | | | | | | | | | |
|  | | | | | | | | | | | | | | | |
| **Other disease genes associated with dystonia and/or optic atrophy** | | | | | | | | | | | | | | | |
| ***AUH****^6,7^* | AR | Variably present | Choreoathetoid movements variably present | Variable (spasticity, quadriplegia) | | Yes (gait and dysarthria) | | No | | Yes (variably present) | Mental retardation | Optic atrophy | Diffuse white matter lesions or basal ganglia involvement |  |  |
| ***TSFM^8^*** | AR | Dystonic posturing reported | Tremor, hyperkinetic movement disorder | No | | Yes | | Axonal sensorimotor neuropathy variably present | | Variably present | Global developmental delay | Optic atrophy/ neuropathy | Basal ganglia involvement |  | Dilated cardiomyopathy |
| ***MT-ND6****^9^* |  | Alone or in association with pathogenic MT-ND4 point mutation^10^ | Tremor, myoclonus | | No | | Variable | Muscle atrophy | Yes | | Psychomotor delay | Leber hereditary optic neuropathy | Typical Leigh syndrome pattern |  | No |
| ***AFG3L2****^11^* | AD/AR | Rare | Myoclonus, parkinsonism in concurrence with SPG7 mutation | | Spasticity | | Main phenotype | Variably present | Homozygous mutations linked to intractable seizures. | | Homozygous mutations linked to regression of developmental milestones, heterozygous associated with mild ID | Isolated or syndromic | Variable, from cerebellar atrophy to basal ganglia involvement, “eye-of-the tiger” sign. |  | Uncommon |
| ***DNM1L****^12,13^* | AD/AR | In few de novo, dystonia | Myoclonus | | Mild spasticity in one case | | Yes. de novo | In few de novo, muscle atrophy and sensory neuropathy | De novo, also status epilepticus | | Mild to moderate developmental delay | De Novo missense variant isolated optic atrophy | Variable involvement of basal ganglia, thalamus, hippocampus, frontal, and temporal lobe. |  |  |
| ***MFF****^14^* | AR | Dystonia of the foot | No | | Spasticity | | No | Muscle weakness | Rare myoclonic seizure | | Developmental delay | Temporal disc pallor | Cerebellar atrophy, basal ganglia involvement | Yes |  |
| ***OPA1****^15^* | AR/AD | In one case cervical dystonia | Syndromic parkinsonism | | Spasticity | | Yes | Mitochondrial myopathy and peripheral neuropathy | Rare, focal occipital epilepsy | | Developmental delay or dementia | Bilateral optic atrophy, external ophthalmoplegia. | White matter abnormalities | Deafness |  |
| ***OPA3****^15-17^* | AR/AD | Variably present | Choreatetosis, extrapyramidal signs, variably present | | Pyramidal sign always present, spasticity | | Dysarthria, gait, and limb ataxia. | Variably present, axonal peripheral neuropathy | Uncommon | | Not usually impaired, but possible developmental delay or regression | Bilateral optic disc atrophy, nystagmus | Chiasmal thinning and cerebellar atrophy | Yes | Gastrointestinal dysmotility |
| ***SPG7****^18^* | AR | Rare | Rare | | Spasticity, Babinski | | Cerebellar ataxia | Muscle weakness | No | | Rare | Rare | Cerebellar atrophy in half cases |  | Bladder and anal sphincter dysfunction |
| ***ATAD3A****^19,20^* | AD, AR | Reported | No | | Spastic paraplegia in some cases | | Cerebellar ataxia | Muscle wasting in some cases | Uncommon | | Variably present developmental delay, from mild to severe | Congenital cataracts, variable presence of optic atrophy | Pontocerebellar hypoplasia |  | Hypertrophic cardiomyopathy |
| ***FDXR****^21^* | AR | Uncommon | Involuntary movements | | Variably present spasticity | | Variably present | Axial weakness, uncommon neuropathy | Uncommon | | Common developmental delay/ regression | Optic atrophy, cataracts | Cerebral atrophy, basal ganglia involvement, delayed myelination | Acoustic neuropathy | Microcephaly and facial dysmorphisms |
| ***POLG****^22^* | AR/AD | Rarely | Parkinsonism | | Rare | | Sensory or cerebellar ataxia, variably present | Common muscle weakness, peripheral neuropathy, less frequent isolated distal myopathy | Uncommon in neonatal onset phenotypes, common intractable seizure in childhood onset , RHADS on EEG | | Common developmental delay or developmental regression | Uncommon Cataracts , ophthalmoplegia, | Stroke-like lesions, less commonly thalamic, basal ganglia and cerebellar involvement | Yes | Hepatic impairment with liver failure, renal dysfunction |
| ***ALG3****^23,24^* | AR | Variably present | No | | Rare | | No | Muscular hypotonia | Common | | Frequent developmental delay | Common, including strabismus and optic atrophy | Cerebral anomalies (dysmorphic hemisphere, dysplasia, atrophy, rarely Dandy Walker) | Yes | Craniofacial abnormalities, feeding problems , cardiac anomalies. |
| ***DNAJC19****^25^* | AR | Variably present | Variably present | | Variably present | | Non progressive cerebellar ataxia | Variably present muscular atrophy | Rare/ uncommon | | Variably present | Variably present | Bilateral basal ganglia involvement or cerebellar atrophy | Yes | Dilated cardiomyopathy, facial dysmorphisms |
| ***FXN****^26^* | AR | Rare laringeal dystonia | No | | Extensor plantar responses | | Frequent ataxia, dysarthia | Muscle weakness, sensory neuropathy | No | | No | Optic tract atrophy | Cerebellar atrophy | Deafness | Foot deformities |
| ***NDUFS3****^27^* | AR | Axial dystonia in few cases | No | | Pyramidalsigns | | Yes | Muscle hypotonia | Febrile seizures | | Yes | Optic atrophy | High T2 signal intensity in the putamen, the white matter, and the brain stem |  | Dysphagia |
| ***MTPAP****^28,29^* | AR | Rarely dystonic posturing | No | | Spasticity | | Spastic ataxia | No | No | | Lethal encephalopathy in 3 cases | Optic atrophy | Diffuse T2 white matter hyperintensities |  |  |
| ***NDUFS2****^30^* | AR | Rarely dystonic posturing | No | | Pyramidal signs | | No | Muscle hypotonia | No | | Developmental regression, uncommon | Optic neuropathy common | Leukodystrophy, brain atrophy |  | Cardiomyopathy |
| ***SLC25A462****^31-34^* | AR | Cervical dystonia reported | Tremor and myoclonus variably present, also associated with Parkinson. | | Rare | | Gait ataxia | Motor and/or sensory neuropathy | No | | Uncommon, associated with Pontocerebellar Hypoplasia type 1 | Bilateral optic atrophy | Cerebellar atrophy and diffuse cerebellar hyperintensities, but in some cases MRI normal |  | Probably associated with Hashimoto’s thyroiditis |

1. Remiche G, Vandernoot I, Sadeghi-Meibodi N, Desmyter L. SPG43 and ALS-like syndrome in the same family due to compound heterozygous mutations of the C19orf12 gene: a case description and brief review. Neurogenetics. 2021 Mar;22(1):95-101. doi: 10.1007/s10048-020-00631-4. Epub 2021 Jan 4. PMID: 33394258.
2. Heimer G, Gregory A, Hogarth P, Hayflick S, Ben Zeev B. MECR-Related Neurologic Disorder. 2019 May 9. In: Adam MP, Ardinger HH, Pagon RA, Wallace SE, Bean LJH, Mirzaa G, Amemiya A, editors. GeneReviews® [Internet]. Seattle (WA): University of Washington, Seattle; 1993–2021. PMID: 31070877.
3. Ha AD, Parratt KL, Rendtorff ND, Lodahl M, Ng K, Rowe DB, Sue CM, Hayes MW, Tranebjaerg L, Fung VS. The phenotypic spectrum of dystonia in Mohr-Tranebjaerg syndrome. Mov Disord. 2012 Jul;27(8):1034-40. doi: 10.1002/mds.25033. Epub 2012 Jun 26. PMID: 22736418.
4. Björkman K, Sofou K, Darin N, Holme E, Kollberg G, Asin-Cayuela J, Holmberg Dahle KM, Oldfors A, Moslemi AR, Tulinius M. Broad phenotypic variability in patients with complex I deficiency due to mutations in NDUFS1 and NDUFV1. Mitochondrion. 2015 Mar;21:33-40. doi: 10.1016/j.mito.2015.01.003. Epub 2015 Jan 20. PMID: 25615419.
5. Zehavi Y, Saada A, Jabaly-Habib H, Dessau M, Shaag A, Elpeleg O, Spiegel R. A novel de novo heterozygous pathogenic variant in the SDHA gene results in childhood onset bilateral optic atrophy and cognitive impairment. Metab Brain Dis. 2021 Apr;36(4):581-588. doi: 10.1007/s11011-021-00671-1. Epub 2021 Jan 20. PMID: 33471299.
6. Wortmann SB, Kluijtmans LA, Engelke UF, Wevers RA, Morava E. The 3-methylglutaconic acidurias: what's new?. J Inherit Metab Dis. 2012;35(1):13-22. doi:10.1007/s10545-010-9210-7
7. Tavasoli AR, Shervin Badv R, Zschocke J, Ashrafi MR, Rostami P. Early infantile presentation of 3-methylglutaconic aciduria type 1 with a novel mutation in AUH gene: A case report and literature review. Brain Dev. 2017 Sep;39(8):714-716. doi: 10.1016/j.braindev.2017.04.007. Epub 2017 Apr 21. PMID: 28438368.
8. van Riesen AK, Biskup S, Kühn AA, Kaindl AM, van Riesen C. Novel Mutation in the TSFM Gene Causes an Early-Onset Complex Chorea without Basal Ganglia Lesions. Mov Disord Clin Pract. 2021 Feb 5;8(3):453-455. doi: 10.1002/mdc3.13144. PMID: 33816677; PMCID: PMC8015916.
9. Karaarslan C. Leber's Hereditary Optic Neuropathy as a Promising Disease for Gene Therapy Development. Adv Ther. 2019 Dec;36(12):3299-3307. doi: 10.1007/s12325-019-01113-2. Epub 2019 Oct 11. PMID: 31605306; PMCID: PMC6860503.
10. Berardo A, Emmanuele V, Vargas W, Tanji K, Naini A, Hirano M. Leber hereditary optic neuropathy plus dystonia, and transverse myelitis due to double mutations in MT-ND4 and MT-ND6. J Neurol. 2020 Mar;267(3):823-829. doi: 10.1007/s00415-019-09619-z. Epub 2019 Nov 27. PMID: 31776719; PMCID: PMC7362294.
11. Eskandrani A, AlHashem A, Ali ES, AlShahwan S, Tlili K, Hundallah K, Tabarki B. Recessive AFG3L2 Mutation Causes Progressive Microcephaly, Early Onset Seizures, Spasticity, and Basal Ganglia Involvement. Pediatr Neurol. 2017 Jun;71:24-28. doi: 10.1016/j.pediatrneurol.2017.03.019. Epub 2017 Apr 5. PMID: 28449981.
12. Pan Z, Wu TH, Chen C, Peng P, He YW, Yi WZ, Yin F, Peng J. [DNM1L gene variant caused encephalopathy, lethal, due to defective mitochondrial peroxisomal fission 1: three cases report and literature review]. Zhonghua Er Ke Za Zhi. 2021 May 2;59(5):400-406. Chinese. doi: 10.3760/cma.j.cn112140-20200921-00893. PMID: 33902225.
13. Keller N, Paketci C, Edem P, Thiele H, Yis U, Wirth B, Karakaya M. De novo DNM1L variant presenting with severe muscular atrophy, dystonia and sensory neuropathy. Eur J Med Genet. 2021 Feb;64(2):104134. doi: 10.1016/j.ejmg.2020.104134. Epub 2020 Dec 31. PMID: 33387674.
14. Panda I, Ahmad I, Sagar S, Zahra S, Shamim U, Sharma S, Faruq M. Encephalopathy due to defective mitochondrial and peroxisomal fission 2 caused by a novel MFF gene mutation in a young child. Clin Genet. 2020 Jun;97(6):933-937. doi: 10.1111/cge.13740. Epub 2020 Mar 24. PMID: 32181496.
15. Del Dotto V, Fogazza M, Lenaers G, Rugolo M, Carelli V, Zanna C. OPA1: How much do we know to approach therapy? Pharmacol Res. 2018 May;131:199-210. doi: 10.1016/j.phrs.2018.02.018. Epub 2018 Feb 15. PMID: 29454676.
16. Yahalom G, Anikster Y, Huna-Baron R, Hoffmann C, Blumkin L, Lev D, Tsabari R, Nitsan Z, Lerman SF, Ben-Zeev B, Pode-Shakked B, Sofer S, Schweiger A, Lerman-Sagie T, Hassin-Baer S. Costeff syndrome: clinical features and natural history. J Neurol. 2014 Dec;261(12):2275-82. doi: 10.1007/s00415-014-7481-x. Epub 2014 Sep 9. PMID: 25201222.
17. Horga A, Bugiardini E, Manole A, Bremner F, Jaunmuktane Z, Dankwa L, Rebelo AP, Woodward CE, Hargreaves IP, Cortese A, Pittman AM, Brandner S, Polke JM, Pitceathly RDS, Züchner S, Hanna MG, Scherer SS, Houlden H, Reilly MM. Autosomal dominant optic atrophy and cataract "plus" phenotype including axonal neuropathy. Neurol Genet. 2019 Apr 1;5(2):e322. doi: 10.1212/NXG.0000000000000322. PMID: 31119193; PMCID: PMC6501639.
18. van Gassen KL, van der Heijden CD, de Bot ST, den Dunnen WF, van den Berg LH, Verschuuren-Bemelmans CC, Kremer HP, Veldink JH, Kamsteeg EJ, Scheffer H, van de Warrenburg BP. Genotype-phenotype correlations in spastic paraplegia type 7: a study in a large Dutch cohort. Brain. 2012 Oct;135(Pt 10):2994-3004. doi: 10.1093/brain/aws224. Epub 2012 Sep 10. PMID: 22964162.
19. de Heredia ML, Clèries R, Nunes V. Genotypic classification of patients with Wolfram syndrome: insights into the natural history of the disease and correlation with phenotype. Genet Med. 2013 Jul;15(7):497-506. doi: 10.1038/gim.2012.180. Epub 2013 Feb 21. PMID: 23429432.
20. Harel T, Yoon WH, Garone C, Gu S, Coban-Akdemir Z, Eldomery MK, Posey JE, Jhangiani SN, Rosenfeld JA, Cho MT, Fox S, Withers M, Brooks SM, Chiang T, Duraine L, Erdin S, Yuan B, Shao Y, Moussallem E, Lamperti C, Donati MA, Smith JD, McLaughlin HM, Eng CM, Walkiewicz M, Xia F, Pippucci T, Magini P, Seri M, Zeviani M, Hirano M, Hunter JV, Srour M, Zanigni S, Lewis RA, Muzny DM, Lotze TE, Boerwinkle E; Baylor-Hopkins Center for Mendelian Genomics; University of Washington Center for Mendelian Genomics, Gibbs RA, Hickey SE, Graham BH, Yang Y, Buhas D, Martin DM, Potocki L, Graziano C, Bellen HJ, Lupski JR. Recurrent De Novo and Biallelic Variation of ATAD3A, Encoding a Mitochondrial Membrane Protein, Results in Distinct Neurological Syndromes. Am J Hum Genet. 2016 Oct 6;99(4):831-845. doi: 10.1016/j.ajhg.2016.08.007. Epub 2016 Sep 15. PMID: 27640307; PMCID: PMC5065660.
21. Stenton SL, Piekutowska-Abramczuk D, Kulterer L, Kopajtich R, Claeys KG, Ciara E, Eisen J, Płoski R, Pronicka E, Malczyk K, Wagner M, Wortmann SB, Prokisch H. Expanding the clinical and genetic spectrum of FDXR deficiency by functional validation of variants of uncertain significance. Hum Mutat. 2021 Mar;42(3):310-319. doi: 10.1002/humu.24160. Epub 2021 Jan 3. PMID: 33348459.
22. Rahman S, Copeland WC. POLG-related disorders and their neurological manifestations. Nat Rev Neurol. 2019 Jan;15(1):40-52. doi: 10.1038/s41582-018-0101-0. PMID: 30451971.
23. Himmelreich N, Dimitrov B, Geiger V, Zielonka M, Hutter AM, Beedgen L, Hüllen A, Breuer M, Peters V, Thiemann KC, Hoffmann GF, Sinning I, Dupré T, Vuillaumier-Barrot S, Barrey C, Denecke J, Kölfen W, Düker G, Ganschow R, Lentze MJ, Moore S, Seta N, Ziegler A, Thiel C. Novel variants and clinical symptoms in four new ALG3-CDG patients, review of the literature, and identification of AAGRP-ALG3 as a novel ALG3 variant with alanine and glycine-rich N-terminus. Hum Mutat. 2019 Jul;40(7):938-951. doi: 10.1002/humu.23764. Epub 2019 May 8. PMID: 31067009.
24. Alsharhan H, Ng BG, Daniel EJP, Friedman J, Pivnick EK, Al-Hashem A, Faqeih EA, Liu P, Engelhardt NM, Keller KN, Chen J, Mazzeo PA; University of Washington Center for Mendelian Genomics (UW-CMG), Rosenfeld JA, Bamshad MJ, Nickerson DA, Raymond KM, Freeze HH, He M, Edmondson AC, Lam C. Expanding the phenotype, genotype and biochemical knowledge of ALG3-CDG. J Inherit Metab Dis. 2021 Feb 13. doi: 10.1002/jimd.12367. Epub ahead of print. PMID: 33583022.
25. Ucar SK, Mayr JA, Feichtinger RG, Canda E, Çoker M, Wortmann SB. Previously Unreported Biallelic Mutation in DNAJC19: Are Sensorineural Hearing Loss and Basal Ganglia Lesions Additional Features of Dilated Cardiomyopathy and Ataxia (DCMA) Syndrome? JIMD Rep. 2017;35:39-45. doi: 10.1007/8904_2016_23. Epub 2016 Dec 8. PMID: 27928778; PMCID: PMC5585102.
26. Williams CT, De Jesus O. Friedreich Ataxia. 2021 May 19. In: StatPearls [Internet]. Treasure Island (FL): StatPearls Publishing; 2021 Jan–. PMID: 33085346.
27. Rahman S, Thorburn D. Nuclear Gene-Encoded Leigh Syndrome Spectrum Overview. 2015 Oct 1 [updated 2020 Jul 16]. In: Adam MP, Ardinger HH, Pagon RA, Wallace SE, Bean LJH, Mirzaa G, Amemiya A, editors. GeneReviews® [Internet]. Seattle (WA): University of Washington, Seattle; 1993–2021. PMID: 26425749.
28. Crosby AH, Patel H, Chioza BA, Proukakis C, Gurtz K, Patton MA, Sharifi R, Harlalka G, Simpson MA, Dick K, Reed JA, Al-Memar A, Chrzanowska-Lightowlers ZM, Cross HE, Lightowlers RN. Defective mitochondrial mRNA maturation is associated with spastic ataxia. Am J Hum Genet. 2010 Nov 12;87(5):655-60. doi: 10.1016/j.ajhg.2010.09.013. Epub 2010 Oct 21. PMID: 20970105; PMCID: PMC2978972.
29. Van Eyck L, Bruni F, Ronan A, Briggs TA, Roscioli T, Rice GI, Vassallo G, Rodero MP, He L, Taylor RW, Livingston JH, Chrzanowska-Lightowlers ZMA, Crow YJ. Biallelic Mutations in MTPAP Associated with a Lethal Encephalopathy. Neuropediatrics. 2020 Jun;51(3):178-184. doi: 10.1055/s-0039-3400979. Epub 2019 Nov 28. PMID: 31779033.
30. Gerber S, Ding MG, Gérard X, Zwicker K, Zanlonghi X, Rio M, Serre V, Hanein S, Munnich A, Rotig A, Bianchi L, Amati-Bonneau P, Elpeleg O, Kaplan J, Brandt U, Rozet JM. Compound heterozygosity for severe and hypomorphic NDUFS2 mutations cause non-syndromic LHON-like optic neuropathy. J Med Genet. 2017 May;54(5):346-356. doi: 10.1136/jmedgenet-2016-104212. Epub 2016 Dec 28. PMID: 28031252.
31. Raju S, Medarametla S, Boraiah N. Dystonia and Hereditary Motor Sensory Neuropathy 6B Due to SLC25A46 Gene Mutations. Mov Disord Clin Pract. 2021 Jan 29;8(3):480-482. doi: 10.1002/mdc3.13139. PMID: 33816684; PMCID: PMC8015911.
32. Bitetto G, Malaguti MC, Ceravolo R, Monfrini E, Straniero L, Morini A, Di Giacopo R, Frosini D, Palermo G, Biella F, Ronchi D, Duga S, Taroni F, Corti S, Comi GP, Bresolin N, Giometto B, Di Fonzo A. SLC25A46 mutations in patients with Parkinson's Disease and optic atrophy. Parkinsonism Relat Disord. 2020 May;74:1-5. doi: 10.1016/j.parkreldis.2020.03.018. Epub 2020 Apr 2. PMID: 32259769.
33. Sulaiman RA, Patel N, Alsharif H, Arold ST, Alkuraya FS. A novel mutation in SLC25A46 causes optic atrophy and progressive limb spasticity, with no cerebellar atrophy or axonal neuropathy. Clin Genet. 2017 Aug;92(2):230-231. doi: 10.1111/cge.12963. Epub 2017 Mar 30. PMID: 28369803.
34. Ivanov I, Atkinson D, Litvinenko I, Angelova L, Andonova S, Mumdjiev H, Pacheva I, Panova M, Yordanova R, Belovejdov V, Petrova A, Bosheva M, Shmilev T, Savov A, Jordanova A. Pontocerebellar hypoplasia type 1 for the neuropediatrician: Genotype-phenotype correlations and diagnostic guidelines based on new cases and overview of the literature. Eur J Paediatr Neurol. 2018 Jul;22(4):674-681. doi: 10.1016/j.ejpn.2018.03.011. Epub 2018 Apr 3. PMID: 29656927.
